# Delirium is not associated with anticholinergic burden or polypharmacy in older patients on admission to an acute hospital: an observational case control study

**DOI:** 10.1186/s12877-016-0336-9

**Published:** 2016-09-21

**Authors:** Hannah C. Moorey, Sebastian Zaidman, Thomas A. Jackson

**Affiliations:** 1 Institute of Inflammation and Ageing, University of Birmingham, Birmingham, UK; 2 College of Medical and Dental Sciences, University of Birmingham, Birmingham, UK; 3 Department of Geritric Medicine, University Hospitals Birmingham, Queen Elizabeth Hospital, Birmingham, UK

**Keywords:** Delirium, Anticholinergic, Polypharmacy, Acetylcholine, Aged, Risk factor

## Abstract

**Background:**

Older people are commonly prescribed multiple medications, including medications with anticholinergic effects. Polypharmacy and anticholinergic medications may be risk factors for the development of delirium.

**Methods:**

Patients from a medical admission unit who were over 70, with DSM-IV diagnosed delirium and patients without delirium, were investigated. Number of drugs prescribed on admission and anticholinergic burden using two scales (the Anticholinergic Cognitive Burden Scale [ACB] and the Anticholinergic Drug Scale [ADS]) were recorded from electronic prescribing records. The relationship and predictive ability of these were explored.

**Results:**

The sample included 125 patients with DSM-IV diagnosed delirium and 122 patients without delirium. The mean age of the sample was 84.0 years. The median number of drugs prescribed was 7: 79.8 % were prescribed ≥5 drugs and 29.0 % ≥10 drugs. The median ACB score was 1 and the median ADS score was 1.5. 73.4 % of patients had an ACB score of ≥1 and 73.0 % had a ADS score ≥1. There was no association between: number of drugs prescribed, rate of polypharmacy, rate of excessive polypharmacy, ACB score and ADS score, and a diagnosis of delirium on admission. Only acetylcholinesterase inhibitor use predicted delirium (OR 3.86, *p* = 0.04) and the number of drugs prescribed was negatively correlated with age (spearman rho = −0.18, *p* = 0.006).

**Conclusion:**

Neither number of drugs prescribed, polypharmacy or anticholinergic burden were associated with delirium on admission, questioning the clinical usefulness of anticholinergic drug scales. Further research is needed to unpick fully the relationship between, drugs, anticholinergic burden, age, and prevalent delirium in older patients and whether there is any role for these scales in clinical practice.

**Electronic supplementary material:**

The online version of this article (doi:10.1186/s12877-016-0336-9) contains supplementary material, which is available to authorized users.

## Background

Delirium is an acute neuropsychiatric condition characterised by deficits in attention and cognition [1]. Delirium is important because it is common among older people acutely admitted to hospital and is associated with greater mortality, in-hospital falls and new institutionalization [2]. The aetiology of delirium is often multifactorial, however studies have found drugs often account for a significant proportion [3, 4]. Polypharmacy has been indicated as a precipitating factor in the development of delirium [5–7], as has the addition of multiple drugs as an inpatient [8]. Furthermore, the use of specific medications or medication classes have been identified as risk factors for the development of delirium [9]. Psychotropic drugs, particularly those with anticholinergic properties, have been implicated as precipitants [10].

Further investigation into the role of anticholinergic drugs in delirium is important as they are commonly prescribed medications [11], and they are biologically plausible as an aetiological factor in delirium [12]. Reduced cerebral acetylcholine has been hypothesized to be the common final pathway in the development of delirium in response to inflammation [13]. Delirium is associated with an increased risk of incident cognitive impairment [14] similar to that seen in chronic use of anticholinergic drugs [15]. This has led to the development of two methods to measure anticholinergic burden. First, anticholinergic burden can be estimated by measuring serum anticholinergic activity (SAA) in vitro. Second, expert-based anticholinergic drug scales estimate anticholinergic burden by assigning medications a score, based on high- low risk, which are then combined to produce an overall score for a patient. However, evidence of association between SAA and anticholinergic drug scales with delirium is contradictory [16–18]. Anticholinergic drug burden on admission has been associated with an admission with delirium [19] and incident delirium in patients admitted with acute stroke [20]. A rise in anticholinergic drug burden as an inpatient has also been associated with development of incident delirium in palliative patients [21]. However, other studies have found no association between the administration of anticholinergic medications and the development of incident delirium in older patients [22, 23], cancer patients [24] and intensive care patients [25].

Delirium is associated with poor outcomes and a high mortality rate [26] and early identification is important. Identifying patients at risk of delirium early may help improve detection and hence care, but it is not clear if medication identification has a role in this. Further research is needed to clarify which factors or scales are clinically useful in predicting delirium. Therefore, the aim of this study was to determine if anticholinergic drug burden, as measured by simple scales, or polypharmacy, were associated with delirium on admission to hospital.

## Method

The objectives of this study were first to investigate associations between anticholinergic burden, measured through two different drug scales, and the presence of delirium in older people presenting to an acute hospital admission unit. Second, to investigate associations between the number of drugs prescribed pre-admission and the presence of polypharmacy with prevalent delirium in older people presenting to an acute hospital admission unit.

### Study sample

Patients aged 70 years and over, with an unplanned medical admission to a United Kingdom university hospital between March 2013 and November 2014, were screened for delirium. A diagnosis of delirium that met the Diagnostic and Statistical Manual of Mental Disorders (4th ed., text rev.; DSM-IV-TR; American Psychiatric Association, 2000) criteria was then made. This utilized screening with the Confusion Assessment Method (CAM) [27], Abbreviated Mental Test Score (AMTS) [28], the Digit Span test, and a detailed review of the medical notes. An expert geriatrician (TAJ) completed all screening. Potential participants who were unable to communicate because of severe sensory impairment or inability to speak in English were excluded, as were those deemed to be at risk of imminent death.

Participants with delirium were recruited as part of a larger study examining undiagnosed cognitive impairment in people with delirium [29]. Routinely collected data on a matched number of people screened as not having delirium were also collected. These were age, gender and hospital length of stay.

### Drug data collection

Electronic prescribing records were used to collect admission medications and drug reconciliation is completed by pharmacists on the admissions ward to ensure accuracy of admission drug history. Drugs prescribed within 48 h of admission were recorded as pre-admission drugs. Clinical judgement was used to exclude drugs that were likely to be an acute prescription on that admission; by considering the likely indication, route of administration and whether drugs were prescribed on discharge. Both regular and PRN medications were included. Vitamins, topical preparations and optic preparations were included. Nutritional supplements were excluded. Any herbal, complimentary and alternative medicines, or over the counter medications regularly taken by the patient were only included if they were prescribed on admission to hospital. Polypharmacy was defined as 5 or more drugs and excessive polypharmacy as 10 or more drugs. The Anticholinergic Cognitive Burden Scale (ACB) and the Anticholinergic Drug Scale (ADS) was then calculated for each participant based on the recorded pre-admission drugs. The ACB and ADS scores were chosen as they are the most widely available scales. The DBI scale is another available scale, this was not used as it is a less specific measure of anticholinergic activity as it includes all psychotropic drugs.

### Statistical analysis

Data were analysed using IBM SPSS Statistics for Windows (Version 22.0. Armonk, NY: IBM Corp.). Normality of data was assessed using the Shapiro-Wilk test. Differences in demographics, length of stay, number of drugs, polypharmacy, excessive polypharmacy and ACB and ADS scores between the delirium and no-delirium groups was assessed using an unpaired *T*-test, the independent samples Mann–Whitney *U* test, or Chi Squared test as appropriate. Spearman Rank Coefficient was used to assess correlations between continuous variables. Logistic regression was used to generate odds ratios and 95 % confidence intervals with: number of drugs, ACB score, ADS score, anticholinesterase inhibitor use, and polypharmacy, as predictor variables controlling for age as a recognized predictor of delirium. The significance level was set at <0.05.

## Results

Of 1327 patients screened for delirium 228 were diagnosed with delirium. Of the patients without delirium data were collected for 125 patients to form the no-delirium group. Of the 228 diagnosed with delirium 125 were recruited and they formed the delirium group. Four patients from the no-delirium group were excluded as they were already included in the delirium group during a previous admission, giving a total sample of 251 (flow chart available as Additional file 1). The mean age of the sample was 84.0 (SD ±6.60) years. There was no significant difference in age or gender between the two groups. The median length of stay was 10 days (IQR 3–20). Participants in the delirium group stayed in hospital significantly longer than patients in the no-delirium group (15 vs 6 days respectively, *p* < 0.001).

The median number of drugs prescribed was 7 (IQR 5–10). Polypharmacy was present in 79.8 % of the sample and excessive polypharmacy was present in 29.0 %. There was no difference between those with and without delirium in number of drugs prescribed, or proportion of those with polypharmacy.

The median ADS score was 1.5 (IQR 0–3) and the median ACB was 1 (IQR 0–2). The median ACB score was the same in the delirium and no-delirium group. The median ADS score was slightly higher in the no-delirium group, 2, than in the delirium group, 1, but this was not significant (*p* = 0.09) (Table 1).

**Table 1:** Comparing Demographics, Anticholinergic Burden and Polypharmacy between Delirium and No-delirium Groups

	Study Population (*n* = 251)	Delirium (*n* = 125)	No-delirium (*n* = 122)	Significance
Mean Age (years, SD)	83.97 (±6.60)	84.4 (±6.52)	83.54 (±6.67)	*p* = 0.21
Female (%)	67.3	62.4	72.4	*p* = 0.95
Median Length of Stay (days, IQR)	10 (3–20)	15 (6–29)	6 (2–13)	*p* < 0.001
Median drugs prescribed	7.00 (5–10)	7.00 (5–10)	7.00 (5–10)	*p* = 0.72
Polypharmacy (%)	79.8	79.2	80.5	*p* = 0.80
Excessive Polypharmacy (%)	29.0	28.8	29.3	p = 0.94
Median ACB score	1.00 (0–2)	1.00 (0–3)	1.00 (1–2)	*P* = 0.58
ACB score ≥1 (%)	73.4	68.8	78.0	*p* = 0.10
Median ADS score	1.50 (0–3)	1.00 (0–2.5)	2.00 (1–3)	*p* = 0.09
ADS score ≥1 (%)	73.0	67.2	78.9	*p* = 0.39
AChE Inhibitor prescribed (%)	5.6	8.8	2.4	*p* = 0.030

The number of prescribed drugs was negatively correlated with age (spearman rho = −0.176, *p* = 0.006). Acetlycholinesterase inhibitor prescription predicted delirium on admission when controlling for age (OR 3.86, 95 % CI 1.05–14.22, *p* = 0.04). Age, number of drugs, presence of polypharmacy or excessive polypharmacy, ADS and ACB scores did not predict delirium on admission.

## Discussion

This observational case control study has demonstrated no relationship between anticholinergic burden and polypharmacy, with delirium in older people admitted to an acute hospital. Increasing age is actually associated with reduced number of drugs taken, and only taking anticholinesterase inhibitor drugs are associated with delirium. As expected the majority of older patients in this study were taking a large number of medications, median 7 drugs, and 73 % were exposed to at least one medication with anticholinergic effects. We found no relationship between either of the anticholinergic drug scales used in this study, ACB and ADS, and prevalent delirium on admission to hospital. We also found no association between the number of drugs patients were prescribed, or the presence of polypharmacy and prevalent delirium. Use of Acetylcholinesterase inhibitors was associated with delirium. Delirium is more common in patients with dementia [3] and this likely explains the correlation. However, acetylcholinesterase use is a poor proxy measure for dementia. Polypharmacy was negatively correlated with age in this population.

Strengths of the study include robust screening for delirium by an expert against recognized criteria, and the use of electronic records to accurately record admission drugs. Recognized limitations are that the drug history was taken in the acute setting which have been shown to be unreliable [30], but the drug reconciliation by ward based pharmacists, and the retrospective collection of the drug history from the electronic record, should have limited this potential bias. We did also recognize that drug prescription is not a reliable indicator of patient compliance. A further limitation is that due to study design we were not able to compare frailty or co-morbidity between those with and without delirium. Given the convenience nature of the no-delirium group it was not possible to be confident about how representative this group was of all patients without delirium.

This study supports previous work which has shown no association between anticholinergic burden and delirium [22–25]. However other studies have reported an association [19, 20]. Studies have investigated different patient populations and this may in part explain the contrasting results. However, studies with a similar population to this study, older patients admitted to hospital, have also had mixed results [19, 22, 23]. The populations investigated were similar with regards to age. However, the cohort in the current study demonstrates a higher burden of polypharmacy, suggesting the cohort may be frailer and more multi-morbid, and perhaps of greater representation of real world practice.

Contrasting results may also be explained by limitations to the drug scales themselves. First, a number of drugs scales have been developed and there are variations between them. The ACB and ADS scales were used in this study. The only study to investigate a similar patient population and find positive results was by Best et al. [19] and used the Drug Burden Index (DBI) [31]. DBI is currently the only scale to consider drug dose but is also a broader drug scale including other sedative drugs without anticholinergic effects [31]. These differences may explain the contrast in the results.

Second, use of anticholinergic medications is likely to vary between populations and this may be reflected in the development of anticholinergic drug scales [32]. The use of scales in different populations may, therefore, not accurately assess anticholinergic burden. Furthermore, drug scales need to be regularly updated to include any new medications with anticholinergic effects [32]. Third, drug scales have been broadly criticised for oversimplifying a complicated pharmacological issue. For example, all scales use a simple linear additive model, however the risk of anticholinergic effects may not increase in this manner [33]. Also drug scales do not consider underlying patient characteristics including differences in pharmacodynamics, cholinergic reserve and endogenous anticholinergic activity [33]. These characteristics are likely to be even more important in a population of older patients, as investigated in this study.

This study shows a negative correlation between age and the number of drugs prescribed, which is in keeping with a previous report [34]. This may be explained by first, that the oldest old (age ≥85) may be healthier than those who die earlier, and therefore take less medications; a survivor effect. Second, this may be due to deprescribing in the oldest old as medications are deemed clinically unnecessary towards the end of life.

This study raises doubts about the clinical utility of polypharmacy and anticholinergic drug scales, at predicting delirium in older hospital populations. However, further research is needed to clarify this and to investigate other factors, such as frailty, that may predispose some older people with polypharmacy and high anticholinergic drug use to delirium. Further research could also investigate which specific anticholinergic drug scale is most useful in delirium or the development of new scales with fewer limitations. The relationship between delirium, anticholinergic drug scale scores and acetylcholine levels or serum anticholinergic levels in vitro could also be investigated further. This may be particularly important in older people who are more likely to have decreased cholinergic reserve and more variable pharmacodynamics.

## Conclusion

Neither number of drugs prescribed, polypharmacy nor anticholinergic burden were associated with delirium on admission. This study contributes to an existing volume of research which questions the usefulness of anticholinergic drug scales in predicting delirium. Further research is needed to fully unpick the relationship between drugs, anticholinergic burden and prevalent delirium in older patients, with a focus on additional factors which contribute to patient vulnerability to delirium and its complex aetiology.

## Additional file

Additional file 1: Figure S1. Flowchart of selection of study participants. Flowchart demonstrating selection of participants to the delirium and no-delirium groups and the number of patients recruited. The number of patients excluded and not recruited are also displayed and the reason they were not included. (DOCX 30 kb)

## Abbreviations

ACB: The Anticholinergic Cognitive Burden Scale
AChEi: Acetylcholinesterase inhibitors
ADS: The Anticholinergic Drug Scale
AMTS: Abbreviated Mental Test Score
CAM: Confusion Assessment Method
DBI: Drug Burden Index
DSMIV: Diagnostic and Statistical Manual of Mental Disorders, 4th Edition
IQR: Interquartile range
SAA: Serum anticholinergic activity
SD: Standard deviation
