# Supplementary material for: Delirium is not associated with anticholinergic burden or polypharmacy in older patients on admission to an acute hospital: an observational case control study
Source: BMC Geriatr. 2016 Sep 21;16:162. doi: 10.1186/s12877-016-0336-9 (PMC5031270; doi:10.1186/s12877-016-0336-9)
Supplement: Additional file 1: Figure S1. — Flowchart of selection of study participants. Flowchart demonstrating selection of participants to the delirium and no-delirium groups and the number of patients recruited. The number of patients excluded and not recruited are also displayed and the reason they were not included. (DOCX 30 kb) [file 12877_2016_336_MOESM1_ESM.docx]

**Supplemental Figure 1: Flowchart of selection of study participants**

**Total Available**

1668

**Total Screened**

1327 (79.5%)

**Exclusion:**

Ill – 48

Communication - 67

Logistics - 226

**Total diagnosed with delirium**

228(17.2%)

**Fully Recruited**

**Delirium group**

125 Recruited (54.8%)

**Reason for not recruiting (total 79):**

Consultee unavailable: 57

Palliative care: 22

Communication: 15

Consultee declined participation: 2

Previously recruited: 7

Identification

Screening

Diagnosis

Recruitment

**No delirium**

**N=126 comparison group**

N=1205 not involved

**4 previously recruited to study**

**Final N=122**
